# Mapping food system drivers of the double burden of malnutrition using community-based system dynamics: a case study in Peru

**DOI:** 10.1186/s44263-024-00045-6

**Published:** 2024-03-01

**Authors:** Carmen Quinteros-Reyes, Paraskevi Seferidi, Laura Guzman-Abello, Christopher Millett, Antonio Bernabé-Ortiz, Ellis Ballard

**Affiliations:** 1 CRONICAS Center of Excellence in Chronic Diseases, Universidad Peruana Cayetano Heredia, Lima, Peru; 2 Public Health Policy Evaluation Unit, School of Public Health, Imperial College London, London, UK; 3 Department of Design, School of Architecture and Design, Universidad de los Andes, Bogota, Colombia; 4 Comprehensive Health Research Center (CHRC) and Public Health Research Centre, National School of Public Health, NOVA University, Lisbon, Portugal; 5 Social System Design Lab, Brown School at Washington University in St. Louis, St. Louis, USA

**Keywords:** Double burden of malnutrition, Peru, Double-duty actions, Food system, Community-based system dynamics, Group model building

## Abstract

**Background:**

Peru is facing a double burden of malnutrition (DBM), characterized by the co-existence of undernutrition and overnutrition. Double-duty actions that concurrently target common drivers of undernutrition and overnutrition, while ensuring no unintended side effects, are recommended to effectively address the DBM. To understand these complex common mechanisms and design context-specific double-duty actions, there is a need for participatory systems approaches. This paper provides a case study of applying a community-based system dynamics approach to capture stakeholder perspectives of food system drivers of the DBM in two regions in Peru.

**Methods:**

We implemented a multi-stage community-based system dynamics approach, which included processes for research capacity building for systems approaches, and the designing, piloting, and implementation of stakeholder workshops. A total of 36 stakeholders, representing diverse perspectives, participated in five group model building workshops. Stakeholder views are presented in a causal loop diagram that showcases the feedback mechanisms between key food system drivers of overweight and stunting in Peru.

**Results:**

The causal loop diagram highlights that prioritization of undernutrition over overnutrition in the policymaking process, due to Peru’s historically high levels of undernutrition, may undermine action against the DBM. It also describes potential mechanisms of unintended impacts of undernutrition policies on the DBM in Peru, including impacts related to within-family distribution and quality of food provided through food assistance programs, and unintended impacts due to regional dynamics.

**Conclusions:**

This paper highlights the importance of a participatory approach to understand local needs and priorities when recommending double-duty actions in Peru and shares practical methodological guidance on applying participatory systems approaches in public health.

**Supplementary Information:**

The online version contains supplementary material available at 10.1186/s44263-024-00045-6.

## Background

Over the last decades, Peru has seen a rapid nutrition transition toward a more obesogenic food system [1, 2], which includes the actors and processes that move food from the farm to the table, their surrounding physical, political and socioeconomic environments, and the subsequent dietary behaviors of consumers [3]. These changes have led to increases in energy consumption from unhealthy foods and concurrent decreases from healthy foods in almost all sociodemographic groups in Peru [4], further leading to rising levels of overnutrition (e.g., obesity and diet-related noncommunicable diseases) [2]. For example, prevalence of overweight among women of reproductive age has increased from 44% in 2005 [5] to 61% in 2021 [6]. At the same time, although undernutrition (e.g., stunting and anemia) has significantly declined since the early 2000s, mainly in urban settings [7], it still persists across regions in Peru with staggering inequalities. For example, in 2021, stunting for children under the age of 5 was 5.3% in Peru’s coastal areas but reached 16.2% and 20.5% in Peru’s jungle and highland regions, respectively [6]. This has led to a double burden of malnutrition (DBM) [8], where overnutrition and undernutrition coexist at the individual (e.g., stunted children with obesity), household (e.g., stunted children living with an obese mother), and population level (e.g., high prevalence of stunting and obesity in the same community) [9]. However, policy action to tackle this emerging problem is lacking [5, 6].

According to the World Health Organization (WHO), efforts to tackle the DBM must consider double-duty actions that require working on common drivers for under- and overnutrition simultaneously [10]. The WHO advises that this can be achieved by designing and implementing nutrition policy in three different ways: (1) assessing and ensuring that current policies that focus on one form of malnutrition do not increase other forms of malnutrition, (2) improving existing interventions by adding solutions that target common drivers of different forms of malnutrition, and (3) creating new double-duty actions to address DBM [11]. Effective implementation of double-duty actions requires understanding the pathways of how policies and changes in the food system have contributed to the DBM in Peru over time. However, the literature on interventions addressing DBM is scarce, with no study explicitly mentioning DBM as a concept of interest [12]. In Peru, no double-duty actions have been enacted, although specific programs that focused on one side of malnutrition have evidenced unintended consequences on other forms of malnutrition [13].

To develop contextually relevant and locally tailored double-duty actions, there is a need for new approaches that investigate common drivers associated with over- and under-nutrition simultaneously and examine how they interact with each other in Peru. This reflects a larger trend in population health research and policy toward advancing systems approaches that are able to embrace the multi-level and multi-disciplinary nature of whole-of-systems health transformations [14, 15]. This argument was reinforced in the 2019 Lancet Commission on Obesity Report, which called for systems approaches to conceptualize and model potential scenarios to address the syndemic of obesity, undernutrition, and climate change [16]. A range of systems approaches [17] has been proposed to complement traditional epidemiological methods for supporting research and policy to address nutrition issues, including agent-based modeling, discrete event simulation, social network analysis, and system dynamics modeling [18, 19]. Yet, a recent review of systems science approaches to obesity and diet found that few applications of these systems science methods have been developed in Latin America, and fewer that specifically focus on participation of multiple stakeholders who experience the realities of DBM [19].

System dynamics (SD) is a systems science method that uses qualitative diagrams and mathematical simulation to understand the way a system is organized in terms of its accumulations, self-reinforcing or self-balancing dynamics, and non-linearities to produce a complex system behavior [20]. Community-based system dynamics (CBSD) is a participatory SD approach that involves community to build consensus of how a complex system is structured and to generate plans for action using a feedback perspective [21, 22]. A feature of the CBSD approach is the use of group model building workshops to convene key stakeholders to build visual representations of system structures through the use of causal loop diagrams (CLDs). CLDs represent a structural hypothesis of the components, their connections, and feedback loops that may explain why a system behaves as it does. Qualitative outputs are often used on their own as tools for dialog, theory generation, or planning. These qualitative diagrams can also be used to develop quantitative simulation models to evaluate how the system may respond to “what if?” scenarios related to specific policy levers [22]. CBSD and other group model building approaches have been previously applied to similar issues like diet [23], public health issues in Latin America [24–26], and food systems [27–31].

To address the methodological and empirical gap around the DBM, a multi-national and transdisciplinary research team has endeavored to conceptualize interconnections between food system components and the DBM in Peru from a feedback perspective, and to identify potential effective policies to address it, using a CBSD approach. This current manuscript presents a case study that aims to describe the application of CBSD to capture stakeholder perspectives on food system drivers of the DBM. It can serve as an exemplar of applying systems approaches in population health.

## Methods

### Study setting and participants

This research focused engagement and modeling in two diverse regions in Peru in order to capture settings with varied DBM characteristics: Lima, the highly urbanized and densely developed capital city located on the coast, with low stunting rates but with one of the highest rates of overweight and obesity in the country; and Iquitos (Loreto region), a low density, regional capital city located in the Amazon jungle, with one of the highest rates of stunting, and overweight and obesity in the country [6]. Within these two localities, the research team conceptualized a broad definition of community stakeholders, comprising parents of young children, representatives of community non-governmental and advocacy organizations, local and regional policymakers, and research community members working on issues of nutrition and public health in Peru. Stakeholders were recruited between February and April 2022.

### Project design

Investigators from Universidad Peruana Cayetano Heredia (Peru) (*n* = 2), Imperial College London (UK) (*n* = 1), Washington University in St. Louis Social System Design Lab (USA) (*n* = 1), and Universidad de Los Andes (Colombia) (*n* = 1) formed a core modeling team to lead the design and implementation of this CBSD effort, including convening of group model building workshops and subsequent model development. The core modeling team was intentionally convened to comprise multiple disciplinary, substantive, and methodological expertise including epidemiology, nutrition, system dynamics simulation modeling, participatory group model building design and facilitation, Peruvian health policy, and familiarity with community stakeholders and the local policy environment. We ensured that the core modeling team included local representatives that led the recruitment of workshop participants and represented them throughout the process. For workshops conducted in Lima, this role was played by two research team members from Universidad Peruana Cayetano Heredia (Lima, Peru), whereas for the workshop conducted in Iquitos, the core modeling team was additionally joined by a researcher from the Universidad Nacional de la Amazonia Peruana, an institution based in Iquitos, and with great influence in the area. Recruitment was conducted by these members of the core modeling team utilizing and further expanding an established network of relevant stakeholders, developed and maintained by the two local institutions through long-standing collaborations in previous projects related to public health.

**Fig. 1:**
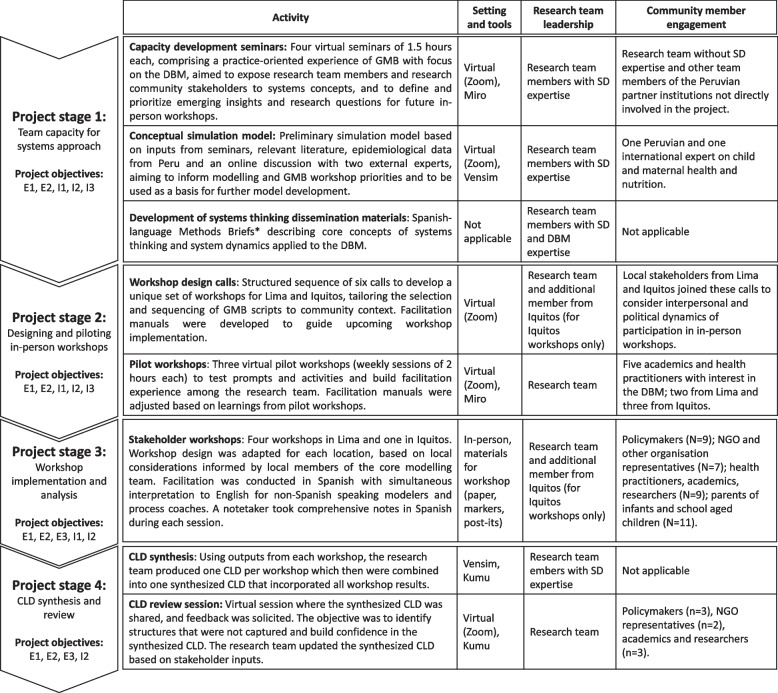
Project stages and activities. SD, system dynamics; GMB, group model building; DBM, double burden of malnutrition. *Methods Briefs are freely available in Spanish and English at the following link: https://socialsystemdesignlab.wustl.edu/publications/methods-briefs/

Though there is a large body of materials and resources for facilitation of group model building workshops, this project required explicit design and formative work to tailor to its specific needs. These included varying levels of familiarity with context and methods across core modeling team members, traveling and in-person meeting restrictions due to the COVID-19 pandemic, and flexibility in planning to accommodate uncertainties around COVID-19 restrictions and emerging political instability in Peru. With these constraints in mind, the core modeling team established a set of explicit and implicit workshop objectives to inform the CBSD design (Table 1) and developed a three-stage approach to (1) develop team capacity for systems modeling and participatory approaches; (2) design and pilot in-person workshops; (3) implement stakeholder workshops; and (4) synthesize and review the CLD. This approach and the activities, tools, dissemination materials (Methods Briefs; https://socialsystemdesignlab.wustl.edu/publications/methods-briefs/), research team involvement, and community member participation of each project stage are presented in detail in Fig. 1.

**Table 1:** Objectives of the community-based system dynamics (CBSD) workshops

**Explicit objectives**
E1: Map the drivers of the food system that contribute to the double burden of malnutrition in two diverse regions in Peru using a CBSD approach
E2: Orient relevant stakeholders to a systems perspective of the double burden of malnutrition
E3: Identify potential policy levers that could address the double burden of malnutrition through changes in the food system to be explored in future simulation analysis
**Implicit objectives**
I1: Build group model building capabilities within the project research team to facilitate community workshops
I2: Support the development of a cohort of community stakeholders with exposure to system dynamics approaches to continue involvement in the project moving forward
I3: Generate resources to replicate application of the CBSD approach in relevant future work

### Analysis

#### Data compilation

A unique feature of the CBSD approach is the rapid creation of an archive of participant-generated artifacts as part of the group model building process. The artifacts are tangible products collected from the different group model building scripts that simultaneously serve as data on participant perceptions of factors or structures relevant to the problem of interest, as well as inputs to subsequent modeling activities [32]. Artifacts from workshops included variables in text form and in the form of behavior over time graphs; thematic grouping summaries of clustered variables and graphs; participant votes generated using stickers; connection circle diagrams; and CLDs. At the close of each workshop, these artifacts were carefully photo documented and compiled to preserve this archive and assure validity and reliability of generated data. Additionally, a dedicated notetaker with relevant background in nutrition documented the narratives and selected direct quotes associated with variables and structures presented during the workshop to support recollection of meaning and associated examples during the synthesis process. Relevant guidance on notetaking practices for group model building workshops was provided in advance of the workshops.

#### Causal loop diagram synthesis

CLD synthesis was conducted as an ongoing and iterative activity of the participatory group model building process over multiple steps (Additional file 1: Fig. S1). Initial CLDs were generated from each stakeholder workshop and reviewed for completeness and correspondence to workshop notes. These single-workshop CLDs were an input to building one synthesized CLD that incorporated perspectives of all stakeholder groups. Synthesis was conducted through a multi-stage process, informed by similar work [25, 33], which included (1) listing the identified feedback loops from each single-workshop CLD and determining common ones; (2) aggregating feedback loops that considered a similar dynamic and were identified in multiple single-workshop CLDs; and (3) refining the synthesized CLD using the database of artifacts and inputs from the research team. Following the initial CLD synthesis, the team reconvened participants for a virtual CLD review session to share the synthesis and solicit feedback and revisions. The synthesized CLD was further adapted to incorporated feedback from this session.

## Results

### Stakeholder participation

We hosted a total of five stakeholder workshops. These included four workshops of 2.5 h each in Lima and one full-day (7 h) workshop in Iquitos. The Lima workshops were joined by a total of 23 participants in four separate small groups of similar stakeholders, including policymakers (workshop 1); NGO and other organization representatives (workshop 2); health practitioners, academics, and researchers (workshop 3); and parents of infants and school aged children (workshop 4). The Iquitos workshop was joined by a total of 13 participants based in Iquitos representing multiple stakeholder perspectives of the local context (Table 2). A total of 8 stakeholders attended the virtual CLD synthesis session, including academics (*n* = 3), policymakers (*n* = 3), and NGO representatives (*n* = 2).

**Table 2:** Characteristics of workshop participants

	Workshop 1: policymakers—Lima	Workshop 2: NGOs—Lima	Workshop 3: health practitioners and academics—Lima	Workshop 4: parents—Lima	Workshop 5: Iquitos
**Total *N***	5	5	5	8	13
Male	3	1	1	0	5
Female	2	4	4	8	8
**Stakeholder group**					
Policymakers	5				4
NGO and other organization representatives		5	1		1
Health practitioners, academics, researchers			4		5
Parents of infants and school aged children				8	3

Though the workshops were designed in advance to replicate the same basic format, the team allowed for flexibility of structure to meet to each workshop’s and stakeholder group’s needs and to incorporate learnings from previous workshops. An overview of activities and script and structure variations are presented in Table 3, and facilitation manuals developed to guide workshops are freely available online [34, 35].

**Table 3:** Workshop activities and structure variations

Activity	Script variation	Description	Workshop 1 Lima	Workshop 2 Lima	Workshop 3 Lima	Workshop 4 Lima	Workshop 5 Iquitos
**Welcome and introduction**		Presentation of an overview of the project and the objectives of the workshop. Brief introduction of the research team and participants using activities to create bonds within the group	X	X	X	X	X
**Introduction to key concepts**		Presentation of the concept of the DBM and the trends overtime of this problem in Peru. Brief introduction of outputs from formative work. Brief explanation of the system thinking iceberg as a tool to introduce community-based system dynamics approach to solve complex problems like DBM	X	X	X	X	X
**Variable elicitation**	Variable elicitation	A facilitator asked prompt questions to participants to elicit food system drivers, which contribute to overweight and/or stunting in Peru, based on their perspectives and experience. Participants shared with the group the factors they considered the most important				X	
	Variable elicitation in lists	A facilitator asked prompt questions to participants to elicit food system drivers, which contribute to, first, overweight and, second, stunting in Peru, based on their perspectives and experience. Participants shared with the group the factors they considered most important, organized in two separate lists for overweight drivers and stunting drivers			X		
	Venn-diagram elicitation	A facilitator asked prompt questions to participants to elicit food system drivers, which contribute to, first, overweight and, second, stunting in Peru, based on their perspectives and experience. Participants shared with the group the factors they considered most important and were prompted to situate them within a Venn diagram that represented stunting, overweight, and their intersection, based on their understanding of the variable as a driver of overweight, stunting, or both. This script can be used to elicit shared variables for dual problems, like DBM					X
	Graphs over time	Participants were prompted to draw a graph that described the behavior over time of food system drivers, which contribute to overweight and/or stunting in Peru, based on their perspectives and experience. First, the facilitator used an example of a variable to create a graph that helped to understand its behavior over a period of time, in a visual way. Participants were then prompted to draw their own graphs over time, choosing the variables they considered the most important, with at least one related to overweight and one to stunting. They, then, shared a description of their graph, encouraged to explain how changes in the variable were linked to changes in overweight and/or stunting	X	X			X
	Dots	Participants were prompted to vote which food system drivers where most important in contributing to overweight and/or stunting in Peru, by adding stickers next to elicited variables or graphs over time			X	X	X
**Structure elicitation**	Connection circles	A facilitator provided an example of the exercise using 2 previously elicited variables. Participants were then asked to choose a few of the previously elicited variables, write them around the perimeter of a circle, and draw how they believe they are interconnected by adding arrows with polarities and additional variables where necessary. Participants worked in small groups and shared their connection circles with the wider group at the end			X		X
	Small group causal loop diagram	A modeler introduced the concept of CLDs and relevant notions, such as polarities and balancing and reinforcing feedback loops, using examples from previously elicited variables. Participants worked into small groups to draw their own CLDs using variables they identified in previous activities, encouraged to add more if needed. Each small group presented their CLD with the wider group				X	
	Large group causal loop diagram	A modeler introduced the concept of CLDs and relevant notions, such as polarities and balancing and reinforcing feedback loops, using examples from previously elicited variables. The modeler then guided participants to further build on the CLD, using previously elicited variables, encouraged to add more if needed	X	X			X
**Reflections and closing**		A facilitator provided reflections about insights gained during the workshop and how they contribute to the overall project, discussed next steps, and acknowledged stakeholders for their participation	X	X	X	X	X

Workshop 1 was attended by policymakers; workshop 2 by NGO and other organization representatives; workshop 3 by health practitioners, academics, and researchers; workshop 4 by parents of infants and school aged children; and workshop 5 by a diverse group of all types of stakeholders

*CLD* causal loop diagram

### Causal loop diagram overview

**Fig. 2:**
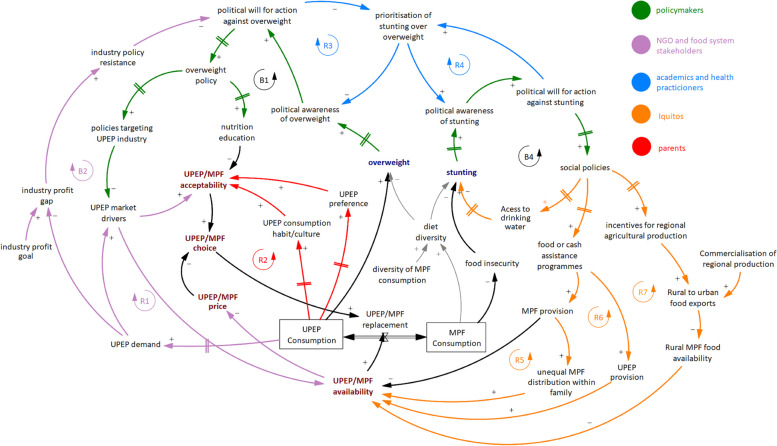
Synthesized causal loop diagram of the food system and the double burden of malnutrition in Lima and Iquitos. The colors represent dynamics that were particularly emphasized by different groups of stakeholders, as noted in the legend. The gray color represents dynamics that were added during the review session. Black arrows were added by the research team during the causal loop diagram synthesis. A Spanish language version of the causal loop diagram and further orientation to reading it is available online [37]. UPEP, ultra-processed edible products; MPF, minimally processed foods

The synthesized CLD captures diverse stakeholder views on food system drivers of overweight and stunting in two regions in Peru, Lima and Iquitos (Fig. 2). The different colors represent dynamics that were particularly emphasized by different groups of stakeholders. CLDs can be read by exploring directed arrows that represent hypothesized pathways of influence between variables. Those arrows include polarities that represent the nature of influence, i.e., positive (+) when the variable relationship operates in the same direction and negative (-) when the variable relationship operates in an opposite direction. Some arrows have parallel marks that indicate that the effect of one variable on another takes longer time (“time delay”). Arrows are combined to form feedback loops that describe structural push and pull on a system. Loops can be self-balancing (noted with B; i.e., a loop to resist or counteract the directions of an initial change) or self-reinforcing (noted with R; i.e., a loop to amplify the directions of an initial change) [22, 36]. Finally, this diagram includes a stock and flow structure representing transitions between consumption of minimally processed foods (MPF) being replaced by ultra-processed edible products (UPEP) and vice versa.

Although there are different manifestations of the DBM, workshops focused on overweight and stunting due to its high prevalence and policy relevance in Peru. The synthesized CLD included a total of seven reinforcing (R) and three balancing (B) feedback loops that are characterized by three main themes: (1) market and behavioral factors driving replacement of healthy MPF with UPEP, (2) feedback structures linking public health intervention and prioritization, and (3) unintended consequences of undernutrition related social policies. Subsequent description provides an overview of key mechanisms of these themes represented in the CLD (Fig. 2).

### MPF and UPEP replacement

Stakeholders identified consumption of UPEP over MPF as a driver of both overweight and stunting, with higher UPEP consumption causing higher levels of overweight and lower MPF consumption causing higher levels of stunting through its effect on food insecurity and diet diversity. A ratio of UPEP over MPF is used to represent dynamics affecting replacement between UPEP and MPF to facilitate visualization, while incorporating specific dynamics for UPEP and MPF, when relevant. Stakeholders identified two reinforcing loops that may impact choice of UPEP over MPF. First, the market response feedback loop (R1) shows that increases in UPEP demand can incentivize the industry to increase its production and marketing (i.e., market drivers that further increase availability and acceptability of UPEP), which further reinforce UPEP consumption. Although not explicitly shown in the diagram, these dynamics can also refer to infant formula, which is considered a UPEP. For example, according to workshop participants, increasing acceptability of infant formula can increase maternal insecurity about breastfeeding, which can further increase demand and subsequently marketing and acceptability of infant formula.

Feedback loop R2 highlights the behavior reinforcement of UPEP consumption. The historically accumulating consumption of UPEP leads to UPEP increasingly becoming part of the population’s dietary habits, encouraging a food culture high in UPEP. At the same time, habitual consumption of UPEP increases preference for them, including through changes in taste and through the social status associated with UPEP consumption. This further increases acceptability and thus consumption of UPEP. The two parallel marks in the arrows linking UPEP consumption with habit/culture and preference represent a delay, which highlights that changes in UPEP consumption take time to translate into changes in habit/culture and preferences. This may result in policy resistance, as policies that target UPEP and successfully reduce their consumption in the short term may be less effective in the long term, given that change in food culture takes time to be achieved.

### Policy action and prioritization

Stakeholders identified balancing feedback loops that resist increases in overweight and stunting. Feedback loop B1 describes policy action against overweight. Increasing levels of overweight can increase awareness of the overweight problem and political will for overweight policy action. According to workshop participants, overweight policies in Peru act by targeting UPEP consumption. They involve policies that target UPEP industry and market drivers, including policies to reduce UPEP availability (e.g., UPEP restrictions in school kiosks) and UPEP acceptability (e.g., food warning labels and advertising restrictions). They can also target nutrition education, for example, through health professionals or education campaigns, which aim to reduce acceptability of UPEP and consequently their consumption. At the same time, however, reductions in UPEP consumption and demand due to overweight policy action can also result in a reduction in industry profits, which incentivizes the industry to resist policy action, e.g., through lobbying, reducing political will for overweight policy (B2).

A variety of social policies were identified as balancing against stunting (B3). Increasing levels of stunting in Peru led to increasing awareness of the stunting problem and political will for stunting policy action. Stunting policies can act through different mechanisms. During workshops, stakeholders explicitly mentioned food assistance programs, such as “Vaso de Leche” or “Glass of Milk,” and conditional cash transfer programs, such as “Programa Nacional de Apoyo Directo a los más Pobres – JUNTOS,” which can increase availability and consumption of MPF, as well as policies that increase access to drinking water.

Although policy action against both overweight and stunting exist in Peru, participants noted that due to Peru’s success story in significantly reducing stunting over the last decades, overweight policy is less likely to be prioritized. This results in two reinforcing feedback loops (R3 and R4), which showcase the continuation of allocating resources to successful stunting policies, even when the problem has shifted. For example, prioritization of stunting over overweight may result to less resources allocated toward overweight monitoring and policy evaluation. Although Peru has enacted some bold policies on overnutrition over the last years, if policymakers cannot monitor overweight progress and there is no evidence to support success of these policy actions, political will for further action against overweight may be reduced, which further motivates prioritization of stunting and resists policy action against overweight (R3). Similarly, as stunting policies are evaluated and deemed effective, action against stunting is further reinforced, which leads to further prioritization of stunting over overweight (R4).

### Unintended side effects of policy action

Workshop participants identified unintended consequences of social programs against stunting, which are represented by reinforcing feedback loops R5, R6, and R7. First, they discussed the distribution of food provided by food assistance programs within family members. Although food assistance programs aim to provide food to households with stunted children, often most of this food is offered to working family members instead. This can unintentionally reduce MPF consumption in stunted children, increasing food insecurity, or even driving replacement of MPF with UPEP, which can lead to overweight (R5). Another potential unintended side effect of food or cash assistance programs is the direct provision of UPEP instead of MPF, which can also lead to both stunting and overweight (R6). Participants noted that this can be directly driven by government schemes, such as tax incentives associated with food donation, which encourage food corporations to donate UPEP that end up being consumed by the most vulnerable. Finally, stakeholders from Iquitos described the role of the recent commercialization of regional MPF on their price and consumption outside major cities. Social policies to combat stunting include incentives to increase regional production of local agricultural products. However, the commercialization of these traditional products can lead to exports from less urbanized areas to urban areas and cities, such as Lima. This results in a reduction in availability and increase in price of local MPF, with unintended side effects to local food insecurity and stunting, potentially enhancing existing inequalities.

## Discussion

This paper provides a case study of the design and implementation of a CBSD process to explore the food system drivers of DBM in Peru. It provides a structured approach on formative work, implementation, and analysis and an overview of relevant tools. By implementing the CBSD process, this study highlights new system structures that drive the DBM in Peru, according to relevant stakeholders, and demonstrates possible contributions of applying a participatory complex systems approach to investigate DBM. Finally, it sets a foundation toward identifying double-duty actions by revealing potential mechanisms of unintended side effects and dual benefits.

In this work, we have illuminated practical and design requirements for implementing a CBSD approach with a newly convened, multi-national and transdisciplinary research team. First, the research team should invest in capacity building before engaging with community stakeholders. This includes building facilitation skills through hands-on participation, for example, in pilot seminars and workshops. It also includes participation of local stakeholders in the core modeling team to co-design workshop structure according to participants’ needs and requirements. Second, formative work plays a significant role in familiarizing with the problem to identify key research questions and important system structures. This can be done through relevant literature reviews and preliminary modeling exercises. Third, designing of workshops should allow for flexibility to adapt facilitation plans during or between workshops. Finally, relationship building early on will secure buy-in from community stakeholders and facilitate understanding of the topic through their perspective.

Using a CBSD approach to explore food system drivers of the DBM in Peru allowed the elicitation of system structures that are specific to the Peruvian context. For example, our results reveal that Peru’s nutrition policy priorities due to its historic high levels of stunting may unintendedly contribute to lack of progress in overweight and obesity. This is an archetype behavior often described in system dynamics as “success to the successful,” where initial success justifies continuing allocation of resources toward successful interventions often in expense of competing problems [38]. The CBSD approach also allowed to highlight the important role of delays in action against overweight and stunting. For example, delays between actual changes in overweight and stunting, political awareness, and implementation of action may result in policymakers acting against outdated problems. This is especially relevant in middle-income countries, like Peru, that are seeing rapid changes in the food environment and dietary consumption patterns [4]. Finally, using a feedback perspective allowed us to characterize several unintended side effects of existing actions that if ignored may result in worsening DBM in the country. Our CLD also re-iterates previous findings on DBM drivers commonly targeted by food policies in Peru and LMICs globally, including market drivers and nutrition education targeting availability, acceptability, and prices of foods [4, 39, 40].

This study highlights the role of contextual design decisions in the planning of group model building workshops. A strength of the group model building process is the structured sequence of scripted exercises to advance the model conceptualization and analysis process. Yet in practice, the combination of scripts and the role of participation varies widely between projects [25, 26, 41–43]. Though resources are available to support selection and facilitation of scripts [44–46], many decisions need to be made in the planning process [47–49]. In this study, we sought to illuminate the design logic and contextual decisions for workshop planning that were informed by overall project goals; external conditions (e.g., COVID-19 restrictions and political unrest in Peru); the capabilities and resources of a multi-national research team; and the specific configurations and norms of communities in the two regions in which the project was implemented. We designed different workshop approaches for each type of stakeholder and community context and sought feedback from all stakeholders on model synthesis in a final review session. The approach offered several benefits to the overall project goals. First, it allowed for each workshop to explicitly respond to the expertise of each stakeholder group. For example, workshops with policy stakeholders mainly focused on the mechanisms through which existing nutrition policies impact DBM in Peru, workshops with NGO and other organization stakeholders focused on food market drivers, workshops with academics and practitioners focused on barriers to implementation and effectiveness of policies, and workshops with community members focused on behavioral drivers of consumption and dietary choice. In Iquitos, where diverse local stakeholders were brought together in a single workshop based on guidance from the local member of the core modeling team regarding travel time and local norms, the workshop focused on dynamics of the DBM specific to the region. Additionally, our approach allowed to gradually build a deeper understanding of the problem by emphasizing issues not adequately addressed in earlier workshops, using relevant prompts. This approach to contextual design of group model building projects responds to concerns for replicability raised by Scott et al. [50]. Instead of seeking to build a universal or generalizable sequence to GMB to support building evidence of the effectiveness of this one approach, we instead lean into Lane’s exhortation for system dynamics modelers to be reflective practitioners, designing interventions based on theory and developing a body of case research to better understand the theoretical underpinnings of what works in practice [51].

This paper highlighted that an important step toward achieving double-duty actions in Peru is to break the reinforcing cycle of prioritizing undernutrition over overnutrition action, which stems from historic trends. One way to achieve this is by recognizing the co-existence of overnutrition and undernutrition in the country and establishing DBM as a political priority. This will allow the monitoring and evaluation of dual nutrition outcomes and shift political awareness toward a synergistic approach. Our results also showcase the importance of engaging with local stakeholders to identify community-driven local priorities for double-duty actions that recognize diversity across regions. For example, stakeholders based in Lima, the country’s capital, identified drivers that increase availability of MPF as an important pathway toward reducing the DBM. However, stakeholders outside Lima highlighted that rural to urban export of traditional MPF may unintendedly increase prices and reduce availability of these foods in the more vulnerable rural areas, which have higher levels of stunting and more rapidly increasing levels of overweight [4]. Although existing recommendations for double-duty priorities exist [39], lack of unintended side effects and maximization of impacts of double-duty actions can only be ensured if they are tailored to specific local needs and priorities.

To our knowledge, this is the first study that implemented the CBSD approach to explore the DBM in Peru. It can be used as guidance to implement similar approaches to population health issues elsewhere. However, the study includes important limitations. First, this study engaged with stakeholders in two diverse regions in Peru, Lima and Iquitos. However, this does not fully capture Peru’s diversity, including rural areas and the highlands. Similarly, only a limited number of stakeholders participated in the workshops. However, the scope of this work was not to develop a generalizable, comprehensive map of the food system in Peru, but to illuminate the structural and feedback understanding of the DBM to inform opportunities for double-duty actions. This did not necessarily require engagement of large numbers of stakeholders, but was achieved through participation of local leaders, who are likely to represent the view of their communities, while making diverse perspectives explicit. Second, although the DBM exists in various manifestations, in this work we focused on the co-existence of overweight and stunting, as they are among the most prevalent in the country, are in the top of policy agendas, and represent chronic issues that are significantly driven by food system drivers. However, other types of malnutrition, such as anemia and NCDs, are also prevalent in the country and closely interlinked and should be explored in future work. Finally, although our approach prioritized long-term stakeholder engagement, issues related to COVID-19 restrictions and nationwide transportation strikes at the time of scheduled workshops resulted in a lack of consistency in stakeholder participation throughout all project stages and activities. For example, ensuring participation of all groups of stakeholders in the final review session, which was conducted online and 4 months after the in-person workshops, was a challenge, and not all groups of stakeholders were represented. This resulted in a review session where discussions had a strong focus on which components of the system were missing and how to further expand the CLD, with a limited discussion of whether the synthesized CLD accurately depicts participants’ mental models. Future implementation of the CBSD approach should facilitate solutions to ensure long-term engagement across the same group of stakeholders, while more explicitly laying out the expectations of engagement in the CLD synthesis process.

## Conclusions

A CBSD approach can provide a valuable vehicle into exploring complex issues, including the DBM. It allows the incorporation of diverse views of stakeholders and uses a feedback perspective to explore opportunities for double-duty actions. Outputs of this work have the potential to inform a quantitative SD model that can inform leverage points for double-duty actions in Peru. This work has contributed to the development of CBSD facilitation capabilities within a multidisciplinary and diverse research team, set the foundation for a network of community stakeholders familiarized with systems notions and ideas and their application in understanding the DBM in Peru, and generated tools that can be used for future applications of CBSD in public health research.

## Supplementary Information

**Additional file 1: Figure S1.** Overview of participatory and non-participatory stages of causal loop diagram (CLD) development.

## Data Availability

The data used for this study include workshop notes in Spanish and photographed artifacts. They are available from the corresponding author upon email request (paraskevi.seferidi14@imperial.ac.uk) to protect participant privacy. Materials used during this study include facilitation manuals, which are publicly available online [34, 35], and dissemination materials in Spanish, which are publicly available online at https://socialsystemdesignlab.wustl.edu/publications/methods-briefs/.

## Abbreviations

DBM: Double burden of malnutrition
WHO: World Health Organization
SD: System dynamics
CBSD: Community-based system dynamics
CLD: Causal loop diagram
