# Supplementary material for: Mapping food system drivers of the double burden of malnutrition using community-based system dynamics: a case study in Peru
Source: BMC Glob Public Health. 2024 Mar 1;2:15. doi: 10.1186/s44263-024-00045-6 (PMC11622957; doi:10.1186/s44263-024-00045-6)

Additional File 1

Figure S1: Overview of participatory and non-participatory stages of causal loop diagram (CLD) development.

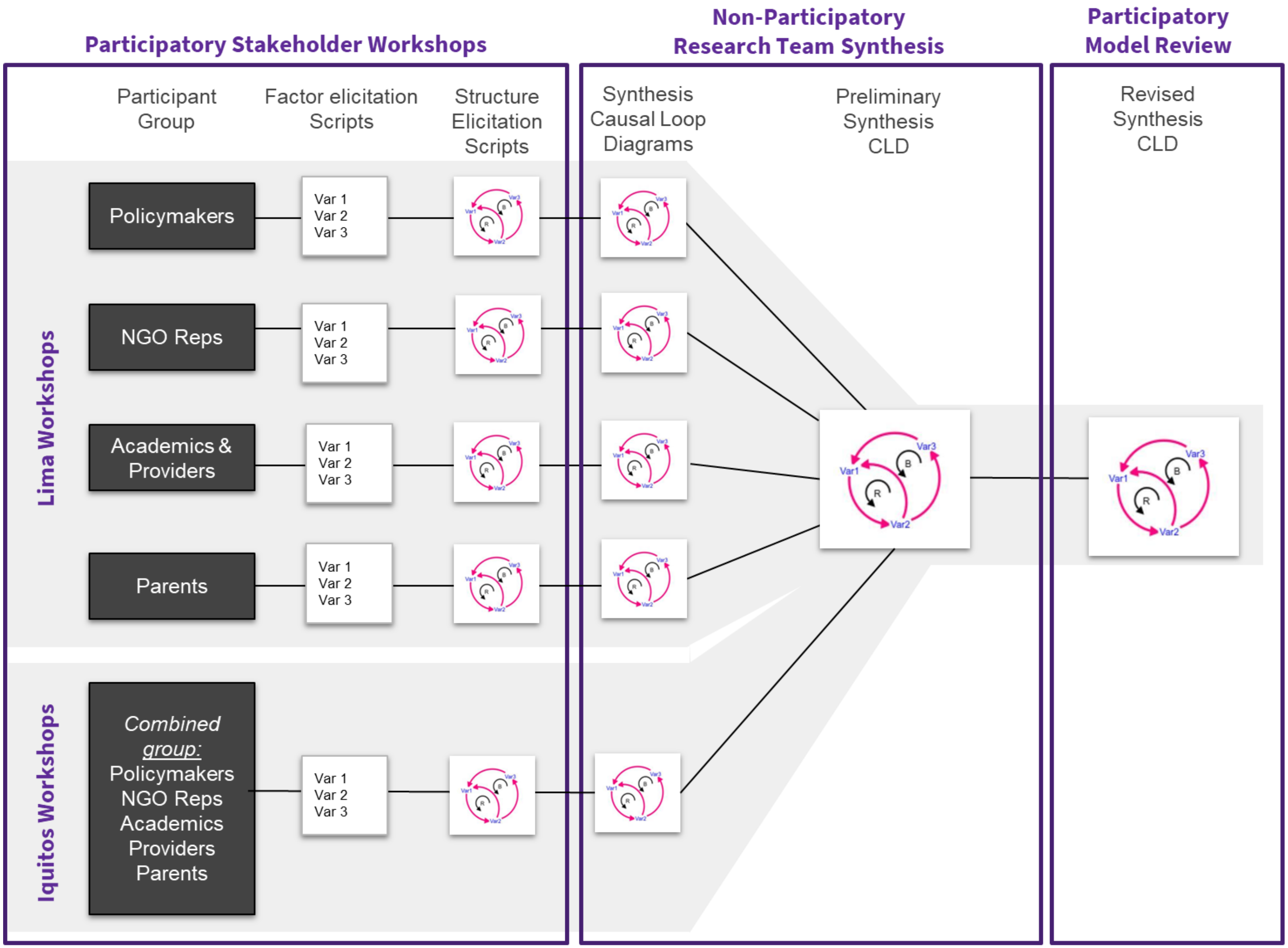

Supplement: Supplementary file 1 — Additional file 1: Figure S1. Overview of participatory and non-participatory stages of causal loop diagram (CLD) development. [file 44263_2024_45_MOESM1_ESM.pdf]
